# The Use of Systemically Absorbed Drugs to Explore An In Vitro Bioequivalence Approach For Comparing Non-Systemically Absorbed Active Pharmaceutical Ingredients in Drug Products For Use in Dogs

**DOI:** 10.1007/s11095-024-03766-3

**Published:** 2024-09-09

**Authors:** Marilyn N. Martinez, Raafat Fahmy, Linge Li, Kithsiri Herath, R. Gary Hollenbeck, Ahmed Ibrahim, Stephen W. Hoag, David Longstaff, Shasha Gao, Michael J. Myers

**Affiliations:** 1 US Food and Drug Administration, Center for Veterinary Medicine, Office of New Animal Drugs, Rockville, MD 20855 US; 2 School of Pharmacy, University Maryland Baltimore, Baltimore, MD 21201 US; 3 US Food and Drug Administration, Center for Veterinary Medicine, Office of Applied Sciences, Laurel, MD 20708 US

**Keywords:** Bioequivalence Evaluation of Non-systemically Absorbed Drugs, In Vivo/in Vitro Relationships, Ivermectin, Praziquantel

## Abstract

**Purpose:**

Currently, for veterinary oral formulations containing one or more active pharmaceutical ingredient (API) that are not systemically absorbed and act locally within the gastrointestinal (GI) tract, the use of terminal clinical endpoint bioequivalence (BE) studies is the only option for evaluating product BE. This investigation explored the use of a totality of evidence approach as an alternative to these terminal studies.

**Methods:**

Three formulations of tablets containing ivermectin plus praziquantel were manufactured to exhibit distinctly different in vitro release characteristics. Because these APIs are highly permeable, plasma drug concentrations served as a biomarker of in vivo dissolution. Tablets were administered to 27 healthy Beagle dogs (3-way crossover) and the rate and extent of exposure of each API for each formulation was compared in a pairwise manner. These results were compared to product relative in vitro dissolution profiles in 3 media. In vivo and in vitro BE predictions were compared.

**Results:**

In vivo/in vitro inconsistencies in product relative performance were observed with both compounds when considering product performance across the 3 dissolution media. Formulation comparisons flagged major differences that could explain this outcome.

**Conclusions:**

The finding of an inconsistent in vivo/in vitro relationship confirmed that in vitro dissolution alone cannot assure product BE for veterinary locally acting GI products. However, when combined with a comparison of product composition and manufacturing method, this totality of evidence approach can successfully alert scientists to potential therapeutic inequivalence, thereby supporting FDA’s efforts to Replace, Reduce, and/or Refine terminal animal studies.

**Supplementary Information:**

The online version contains supplementary material available at 10.1007/s11095-024-03766-3.

## Introduction

For non-systemically absorbed orally administered active pharmaceutical ingredients (APIs) that act locally within the gastrointestinal (GI) tract, alternatives to blood level in vivo bioequivalence (BE) trials are necessary [1]. For many years, the only option for evaluating the BE of products containing locally acting APIs for the treatment of dog and cat GI parasitic infections has been terminal clinical endpoint BE studies. These trials typically include three groups of artificially infected animals: an untreated control, the group receiving the reference product, and the group receiving the test formulation [2]. Because of the high variability associated with these studies, a large number of study animals are often necessary [3].

Opportunities to avoid the need for these terminal clinical endpoint studies are consistent with requirements of the Animal Welfare Act and with the FDA goal to Replace, Reduce, and/or Refine the use of animals in research [4]. In addition, the availability of other approaches to demonstrate BE could avoid the negative long-term ramifications associated with potential future shifts in parasite exposure–response relationships [5]. It has been suggested that in lieu of the clinical endpoint BE trials, comparative in vitro dissolution can serve as the primary evidence of BE for these veterinary products. However, these compounds typically exhibit low aqueous solubility, adding to the complexity of the in vitro test conditions. Alternatively, in vitro dissolution could serve as one component of a totality of evidence approach [6].

A commonality between locally acting and systemically absorbed drugs is the critical step of in vivo drug solubilization/dissolution. For those systemically absorbed drugs that are permeable throughout the GI tract, the in vivo dissolution/drug solubilization is the rate limiting step in drug absorption [7]. For locally acting products, the goal is for equivalence of the exposure of the solubilized drug at the site of the infection. For orally administered, systemically available drugs, it is essential that the test and reference products present equivalent drug concentrations at the site(s) of absorption. Therefore, in an effort to explore the possibility of utilizing in vitro and formulation information to evaluate product equivalence without the need for invasive or terminal clinical endpoint BE experiments, in vivo blood level product BE was used as a “biomarker” for the assessing the equivalence of product in vivo dissolution characteristics. However, for study outcomes to pivot on the comparability of rate and extent of in vivo drug solubilization/dissolution, it was necessary to employ compounds where solubilization/dissolution and not permeability were the rate limiting factors affecting in vivo drug absorption. With this in mind, we explored the relative bioavailability of three laboratory generated tablet formulations containing two poorly soluble, permeable APIs and comparing these in vivo results to formulation and in vitro dissolution differences. The APIs, ivermectin (IVM) and praziquantel (PRZ), were selected because of their differences in pharmacokinetic (PK) and physicochemical characteristics, because drug solubilization/dissolution constitutes the rate limiting step in their oral bioavailabilities, and because this drug combination is contained within an FDA-approved canine oral tablet formulation [8]. In this study, the rate and extent of in vivo drug exposure served as a biomarker for in vivo dissolution and was considered relative to the in vitro tablet performance generated in 3 media.

IVM and PRZ PK information is provided in the Supplemental Material.

## Methods

PRZ (as the racemate) and IVM (total moiety) concentrations were simultaneously measured in the in vivo and the in vitro samples.

A. Formulation development, drug solubility, and in vitro dissolution testing:

Formulation development and in vitro testing of the experimental formulations was conducted at the University of Maryland School of Pharmacy (UMDSP) under contracts F23181018OP and 75F40120C00077 with the CVM/Office of New Animal Drug Evaluation (ONADE). To reflect drug quantities in the FDA-approved combination tablets, all tablets contained 57 mg PRZ and 180 µg IVM. By individually controlling the composition and particle size of the PRZ and IVM granules and then combining the various granules into monolithic tablets, the three formulations exhibited different rates of drug release.

Wet granulation was utilized to produce fast release PRZ (PF), fast release IVM (IF), medium release PRZ (PM) and medium release IVM (IM). The PM and IM granules were intended to slow API release. The nominal composition of these granules is presented in Table 1. Details regarding the composition and manufacture of these tablets are described elsewhere [9].

**Table 1:** Nominal Composition of Fast Release (F) and Medium Release (M) PRZ and IVM Granules

Unit Dose Amounts (mg)				
Granules	IVM^1^		PRZ	
Release Designation	IF	IM	PF	PM
Praziquantel			57	57
Ivermectin	0.2	0.2		
Butylated Hydroxyanisole	0.24	0.24	0.24	0.24
Butylated Hydroxytoluene	0.24	0.24	0.24	0.24
Milled Lactose Monohydrate			108	
Microcrystalline Cellulose	104.3		53.52	
Classified Dibasic Calcium Phosphate Dihydrate (< 150 µm)	120	263.3		200.5
Pregelatinized Starch	75		75	
Hydroxypropylcellulose (HPC)		36		42
Croscarmellose Sodium			6	
Total	300	300	300	300
Sieve Cut	40/60 20/40	20/40	40/60 20/40	18/25
Average drug amount in 300 mg^2^	0.176 0.203	0.203	46.99 48.76	50.47
%Relative Standard Deviation^3^	1.64 1.20	2.01	1.31 1.72	0.98

^1^Target dose was 0.18 mg. A 10% excess was added because of anticipated loss during processing. Batch content uniformity testing confirmed that the targeted amount of IVM was present in each formulation

^2^The bottom 2 rows reflect the average amount of drug added as a function of Sieve Cut and the %RSD associated with the measurement of the amount of drug present

^3^Data based upon 6 replicates per formulation and sieve cut

In addition to formulation, manufacturing differences were also used to create tablets with differing in vitro release profiles. Specifically, Treatment A was made by combining the small particle size granules of the immediate release formulations (i.e., PF sieve cuts = 40/60 and IF sieve cuts = 40/60). Treatment B was made by combining PF (20/40) and IM (20/40) granules so that IVM would be released at a rate slower than that of the tablets containing the IF granules. To slow the release of PRZ, Treatment C consisted of PM granules (sieve cut = 18/20) and IF (sieve cut = 20/40) granules). The rationale for the sieve cut selections is described in the aforementioned manuscript by Hollenbeck et al. [9].

Three hundred (300) mg of PRZ granulation, 300 mg of an IVM granulation, and lubricant were blended and compressed to produce 600 mg tablets containing 57 mg PRZ and 0.18 mg IVM.

- Treatment A: PF/IF
- Treatment B: PF/IM
- Treatment C: PM/IF

Tableting compression was set to 15 kN force. The clinical batches (500–600 tablets/batch) were placed into heat sealed foil pouches to ensure stability and tablet integrity. Tablets were subsequently housed at the CVM Office of Applied Science (OAS) at 25^0^C until completion of the dosing phase.

B. In vitro dissolution

The selection of in vitro dissolution conditions was based upon those described by the USP 29 monographs for IVM [10] (0.01 M phosphate buffer, pH 7, with 0.5% sodium dodecyl sulfate) and PRZ [11] [0.1 N HCl containing 2.0 mg (sodium lauryl sulfate (SLS)]. The assumption is that sodium dodecyl sulfate and SLS are effectively interchangeable and therefore SLS was used across all pH conditions. As these media successfully discriminated between the fast and medium release formulations and were enabled the simultaneous measurement of the rate and extent of PRZ and IVM in vitro dissolution, they were considered appropriate for two of the three dissolution media included in this study. The inclusion of acetate buffer, pH 4.6 + 0.5% SLS was necessary to cover the conditions of the upper small intestine.

**Fig. 1:**
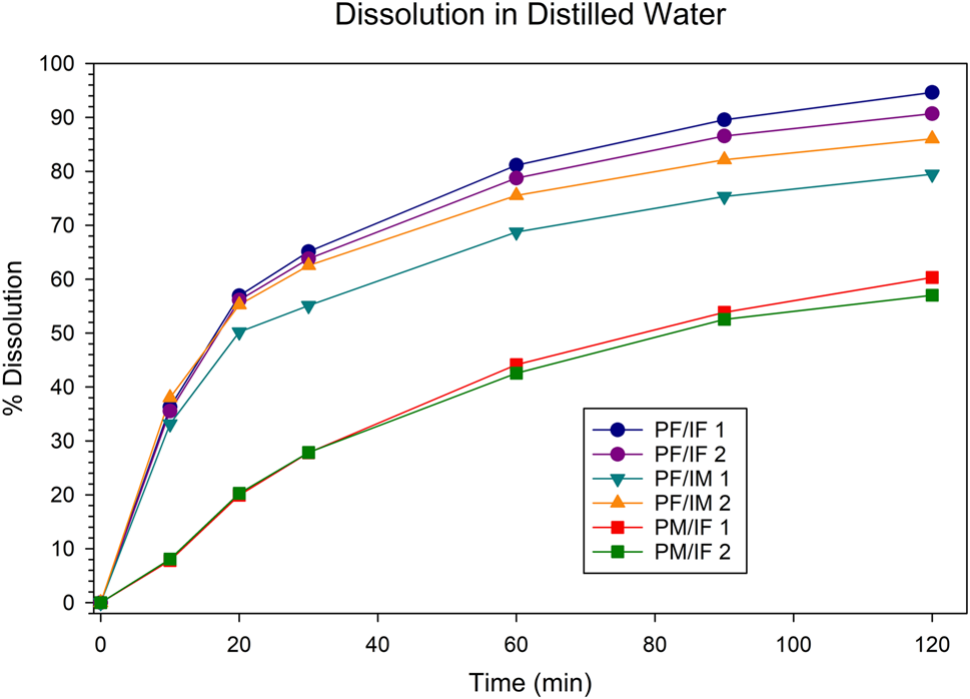
PRZ dissolution profile in 500 mL distilled water, paddle, 50 RPM

The initial dissolution tests were conducted in 500 mL degassed distilled water using apparatus 2 (paddle) and peak vessels at a rate of 50 revolutions minute (RPM). The sample volume was 2.5 mL (*n* = 2 per formulation). The PF/IF tablets disintegrated to granules within 1 min while the tablets containing HPC disintegrated more slowly. Substantial variability in the PRZ dissolution profiles was seen across all formulations, with the extent of release from the PF/IF tablets exceeding that of PF/IM (Fig. 1). Similar results were obtained in human Fasted Simulation Small Intestinal Fluids (results not shown). Since IVM release was not observed in the absence of surfactant from any formulation, all subsequent testing was conducted in media containing SLS.

The pivotal in vitro dissolution tests were conducted in 500 mL buffer, paddle 50 RPM, in the following media:

1. 0.1N HCl + 0.2% SLS (n = 6 per formulation)
2. 0.1 M acetate buffer pH 4.6 + 0.5% SLS (n = 6 per formulation)
3. 0.01 M phosphate buffer, pH 6.8 + 0.5% SLS (n = 12 per formulation)

Release of the APIs in each vessel was determined from a single aliquot of the medium at 10, 20, 30 60, 90, and 120 min.

III. In vitro assay

The UMDSP developed an Ultra-Performance Liquid Chromatography (UPLC) method with diode array detection for simultaneously measuring dissolved concentrations of PRZ and IVM [12]. The method employed gradient elution at a flow rate of 0.7 mL/min using a ternary mixture of water, acetonitrile and methanol. The injection volumes were 10 µL for assay of the tablets (content uniformity) and 50 µL for quantification of drug concentrations in the in vitro dissolution samples. Linearity was indicated for the detector response over the range 57.86 to 636.46 mg/mL for PRZ with an R2 statistic of 0.9996 and for IVM over a range of 0.1821 to 2.003 μg/mL with an R2 of 0.9997. The corresponding accuracy for both compounds ranged between 98.0 and 102.0% with an inter-day precision of less than 2.0% coefficient of variation (CV). The specificity confirmed the absence of interference from tablet excipients, impurities or degradation products. The limit of detection (LOD) and quantitation (LOQ) for IVM was 1.39 μg/mL and 4.22 μg/mL, respectively and for PRZ was 26.80 ng/mL and 81.22 ng /mL, respectively.

IV. Solubility

To ascertain the degree to which SLS and HPC affected IVM and PRZ solubility, drug solubility was determined in water or buffer without HPC or SLS, with HPC, with SLS, or with HPC + SLS. HPC was included in the assessment since it has been suggested to serve as a solubilizing agent [13, 14] and observations that its inclusion (10/100 w/w HPC/drug) can increase the bioavailability of a weak base by reducing the size of precipitated granules [15].

V. In vivo study

The in vivo study was conducted under Good Laboratory Practice (GLP) conditions at the CVM Office of Applied Science (OAS) using a balanced three-sequence, three-period, three-treatment crossover (ABC, BCA, CAB) with an 8-week washout interval between periods. The study employed 27 intact male Beagle dogs, approximately 7 months old, 9 kg ± 1 kg (9 animals per sequence).

An HPLC/MS/MS method was used to simultaneously quantify concentrations of IVM and PRZ in dog plasma. The LOD and LOQ for IVM was 0.17 ng/mL and 0.5 ng/mL, respectively and for PRZ was 1.7 ng/mL and 5 ng /mL, respectively (see Supplemental Material for details). The linear range was established between 0.5 – 500 ng/mL for IVM and 5—5000 ng/mL for PRZ.

Prior to dosing, the dogs were fasted overnight. Water was provided ab libitum. At 12 h postdose, dogs were offered their regular meal. Dogs were pair housed prior to dosing, singly housed during the first 12 h of blood collection and pair housed again after the 12-h blood sample collection. Treatments were orally administered (hr zero) with 10 mL tap water. With the exception of the dosing day, dogs were fed once daily at 8 AM and provided daily socialization, and exercise. Daily observations continued throughout the study.

Blood samples (1 mL each) were collected by direct venipuncture from the cephalic or saphenous veins using a 1 mL or 3 mL syringe with 20 or 22 g needles at 0 h (within 1 h prior to dosing) and at 1, 2, 3, 4, 5, 6, 8, 12, 24, 72, 120, 168, and 240 h post-dose (± 5 min). The blood sampling schedule was based on reported IVM oral PK profiles (IVM time to maximum plasma concentrations, Tmax, in fasted dogs of approximately 4 h postdose and a terminal elimination half-life (T½β) of 3–4 days) [16]. The reported PRZ fasted oral Tmax in dogs ranged across formulations from 0.25 – 1.5 h [17], 1—1.5 h [18], and 2.5 h Yang et al., 2019 [19] and a T½β of approximately 2 h was reported after intravenous injection [20]. Logistic constraints prevented conducting a pilot study to optimize blood sampling times. Therefore, it was assumed that due to inherent variation in PRZ and IVM Tmax values, the proposed sampling schedule would suffice for our BE assessments.

At the end of the in vivo portion of the study, all dogs were adopted as family pets.

F. Data Analysis

A noncompartmental analysis of the plasma concentration versus time profiles was generated using Phoenix 8.3–8.3.5 (2022). Estimated parameters included observed peak concentrations (Cmax), Tmax, area under the concentration versus time curve from time zero to the last time with quantifiable drug concentrations (AUC0-last, linear-log trapezoidal method) and the AUC from time zero to hr 2 or 3 postdose (PRZ and IVM, respectively, linear trapezoidal method). The times included in the partial AUC estimates were based on when the majority of dogs achieved Tmax in all formulations.Statistical analysis of the ln-transformed noncompartmental parameter values were generated in SAS V9.4 using Proc Mixed. The model included treatment, sequence and period as fixed effects, and subject nested in sequence as a random effect. The 90% confidence intervals (CI) for each pairwise comparison was generated using the estimate statement. Although the study is designed as a three-treatment crossover, an adjustment of α was not necessarily due to use of pairwise comparisons [21].

The in vitro dissolution profiles were compared using the f2 metric [22]:

1 $$f2=50log\left\{100{\left[1+\frac{1}{n}\sum_{t=1}^{n}{\left({R}_{t}-{T}_{t}\right)}^{2}\right]}^{-0.5}\right\}$$

where *n* is the number of time points, and *R*_*t*_ and *T*_*t*_ are the mean dissolution (in percent) of the reference and test product at time *t*, respectively.

## Results

A. Solubility

SLS markedly increased the solubility of both APIs. The presence of HPC (a component of all medium release granules) minimally affected IVM and PRZ solubility (Table 2).

**Table 2:** Solubility of IVM and PRZ after 24 h at 37^0^C

	IVM (ug/mL)^*^	PRZ (ug/mL)
Water	1.4	270
Water + 0.2% HPC	8.6	333
Water + 0.5% SLS	4258	1864
Water + 0.2% HPC + 0.5% SLS	4700	1782
0.1N HCl		272
0.1N HCl + 0.2% HPC	8.6	274
0.1 N HCl + 0.2% SLS	20.1	837.8
0.1N HCl + 0.2% HPC + 0.2% SLS	19	563
pH 4.6 Buffer		265
pH 4.6 Buffer + 0.2% HPC	6.4	346
pH 4.6 Buffer + 0.5% SLS	3548	1972
pH 4.6 Buffer + 0.2% HPC + 0.5% SLS	3206	1704
pH 7 Buffer		295
pH 7 Buffer + 0.2% HPC	12.5	287
pH 7 Buffer + 0.5% SLS	6134	1845
pH 7 Buffer + 0.2% HPC + 0.5% SLS	5018	2382

^*^Due to negligible aqueous solubility in the absence of SLS (e.g., as previously discussed for 0.1 N HCl under Results), IVM solubility in the absence of SLS was not determined

In 0.1 N HCl without surfactant, IVM exhibited negligible solubility. This confirmed the need for inclusion of a surfactant for all IVM dissolution studies. However, in 0.1N HCL + 0.2% SLS, the dissolved IVM degraded, as evidenced by a change in sample color from clear to yellow and confirmed chromatographically (Supplement Figs. 1Sa, b).

B. In vitro dissolution:

The mean % dissolved and the %CV for each formulation in each condition and at each timepoint is provided in the Supplemental Table 1S. The f2 results for the IVM and PRZ components are summarized in Table 3.

**Table 3:** Conclusions from *f*2 results for IVM and PRZ

TRT	IVM	PRZ
	**0.1N HCL + 0.2% SLS**	
**A v B**	**Fail (** ***f2*** **= 17.30)**	**Pass (** ***f2*** **=72.89)**
**A v C**	**Fail (** ***f2*** **= 40.28)**	**Fail (** ***f2*** **=38.90)**
**B v C**	**Fail (** ***f2*** **=25.48)**	**Fail (** ***f2*** **=36.82)**
	**0.1 M Acetate buffer pH 4.6 + 0.5% SLS**	
**A v B**	**Fail (** ***f2*** **=20.02)**	**Pass (** ***f2*** **=80.81)**
**A v C**	**Fail (** ***f2*** **=41.36)**	**Fail (** ***f2*** **=26.32)**
**B v C**	**Fail (** ***f2*** **=29.38)**	**Fail (** ***f2*** **=18.92)**
	**Phosphate buffer pH 6.8 + 0.5% SLS**	
**A v B**	**Fail (** ***f2*** **=22.92)**	**Pass (** ***f2=*** **75.26)**
**A v C**	**Fail (** ***f2*** **=46.34)**	**Fail (** ***f2*** **=21.89)**
**B v C**	**Fail (** ***f2*** **=28.49)**	**Fail (** ***f2*** **=23.53)**

1. IVM

The f2 metric was less than 50 for all pairwise comparisons, all conditions.

A decline in dissolved drug in 0.1 N HCl + 0.2% SLS was observed for the Treatments A and C (decline in Treatment A > C), but was not observed for Treatment B. While the solubility chromatograms suggested IVM degradation (rather than precipitation), degradation alone could not explain why this decline was not observed for Treatment B. However, this observation which occurred only in the acidic medium underscores why multiple media are needed to support an in vitro BE approach.

**Fig. 2:**
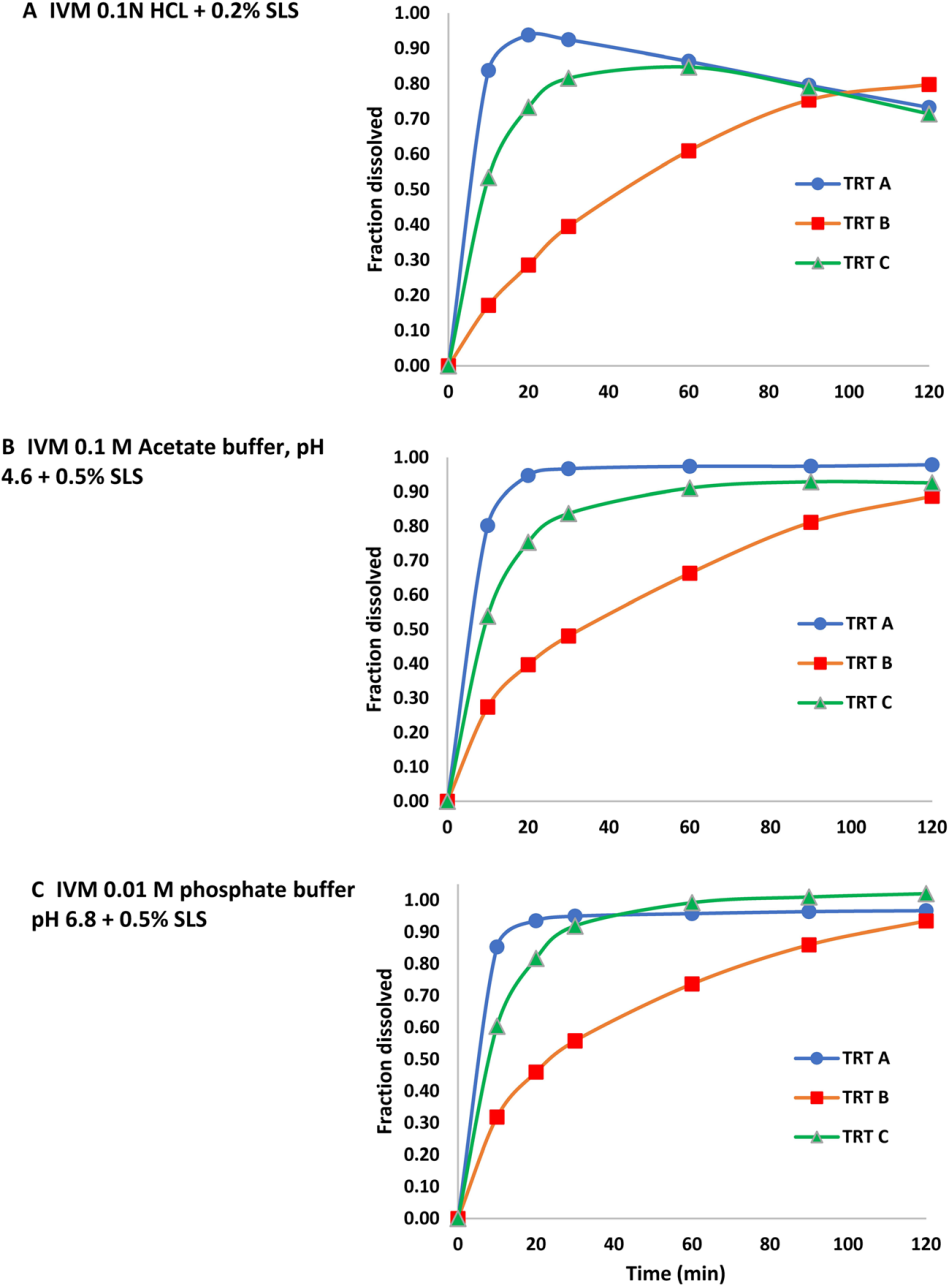
IVM in vitro dissolution profiles under the three sets of test conditions

The mean % dissolved versus time profiles for all treatments and conditions is provided in Fig. 2. Except for 0.1 N HCL + 0.2% SLS, all treatments exhibited > 85% dissolution in 120 min. Greater than 85% dissolution occurred in 20, 120, and 60 min in acetate buffer and 10, 90, and 30 min in phosphate buffer for Treatments A, B, and C, respectively.

2. PRZ

**Fig. 3:**
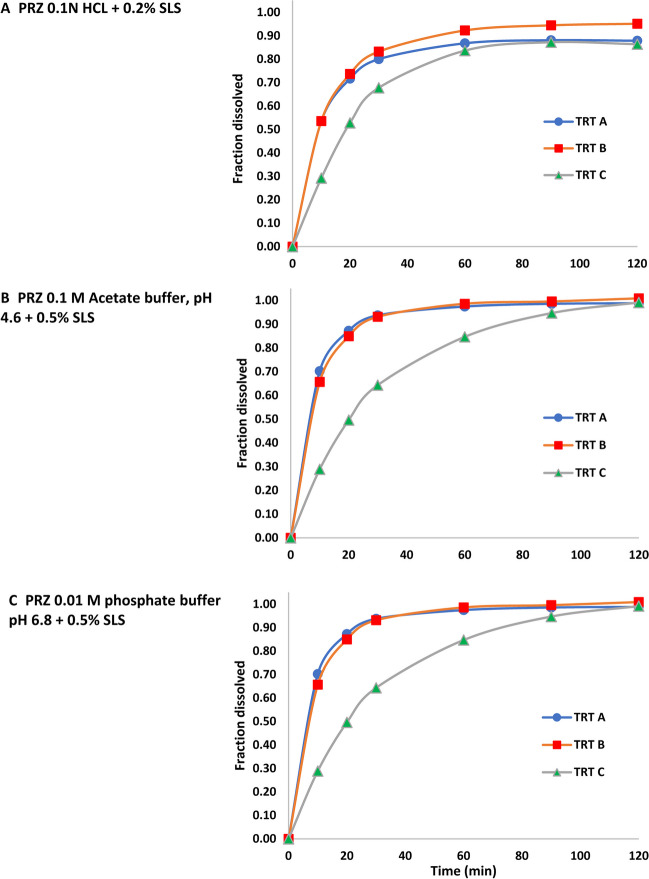
PRZ in vitro dissolution profiles under the three sets of test conditions

Greater than 85% release of the PF formulations (Treatments A and B) occurred within 30 min in 0.1 N HCl + 0.2% SLS and within 20 min in the other two media. The PM formulation (Treatment C) dissolved more slowly, not reaching 85% dissolution for 90 min and 60 min in the acetate and phosphate buffers, respectively. In 0.1 N HCl + 0.2% SLS, Treatment B dissolution was slightly faster than that of Treatment A, but the two treatments were virtually indistinguishable under all other conditions. Treatment C dissolved slowly, failing to meet f2 > 50 for any of the pairwise comparisons under any test conditions (Fig. 3).

A. In vivo results

The absence of IVM or PRZ chromatographic peaks in all hr zero samples confirmed the adequacy of the washout period.

1. IVM

**Fig. 4:**
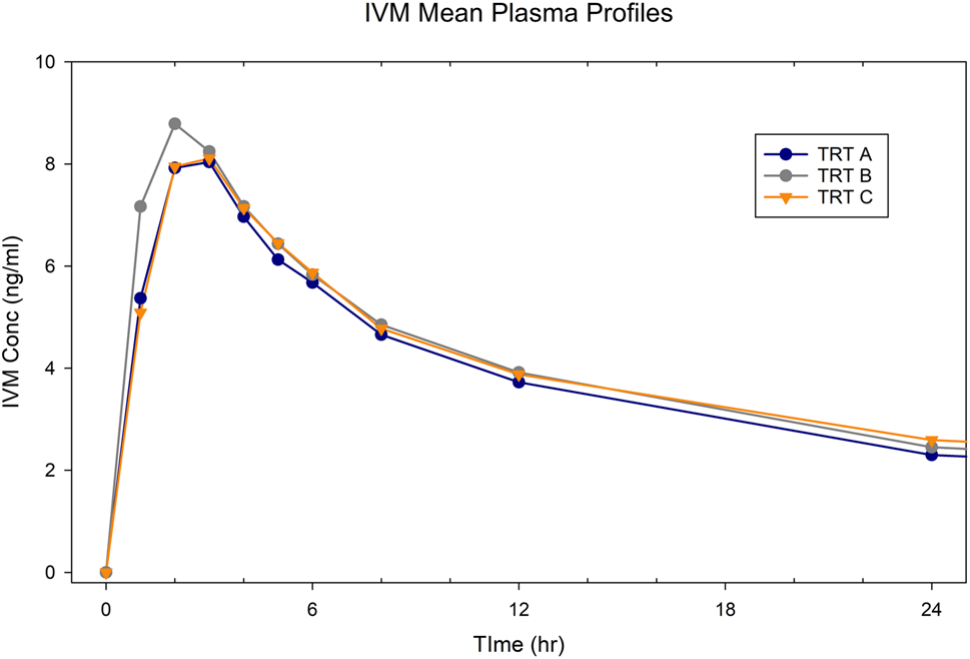
Mean IVM concentration versus time profile up to hr 24 postdose (when all dogs exhibited quantifiable concentrations)

The average profiles (n = 27 dogs, all treatments) are provided in Fig. 4. The parameter geometric means, minimum, maximum, and range values for each Treatment and the 90% CI for each pairwise comparison is provided in Table 1.

**Table:** Table 4

IVM	Exponentiated values		Geometric means		Range of values		
Label	Lower CI	Upper CI	Trt	Geomean	Min	Max	Range
AUC0-last (ng×hr/mL)							
A v B	0.74	1.08	A	137.6	18.16	244.45	226.29
B v C	0.81	1.19	B	153.89	55.07	249.84	194.76
A v C	0.73	1.06	C	156.43	50.67	302.69	252.03
Cmax (ng/mL)							
A v B	0.74	1.02	A	8.14	1.12	16.10	14.98
B v C	0.91	1.26	B	9.37	3.91	12.95	9.04
A v C	0.79	1.09	C	8.79	4.45	16.64	12.19
AUC0-3 (ng×hr/mL)							
A v B	0.67	0.94	A	15.1	2.52	35.45	32.93
B v C	1.01	1.41	B	18.99	8.92	28.80	19.88
A v C	0.80	1.12	C	15.92	6.92	40.04	33.13

Although all pairwise comparisons failed to demonstrate product BE, the in vitro dissolution profiles did not accurately reflect the relative rates and extent of IVM bioavailability. The expectation was that the Cmax, and early AUC values of Treatment A would exceed that of Treatment C and that both A and C would exceed that of Treatment B. However, the Treatment B Cmax, AUC0-last and AUC0-3 values exceeded those of Treatment A, and the Treatment B AUC0-3 values exceeded those of Treatment C. Potentially, the lower rate and extent of exposure for Treatment A (and to some extent C) relative to Treatment B may have been a function of the degradation seen in 0.1 N HCl + 0.2% SLS. This may also explain why Treatments A and C had effectively equivalent Cmax and AUC0-3 values despite the substantially lower AUC0-last of Treatment A. Treatment A consistently had the lowest minimum values for any of the three PK parameters and Treatment B had the smallest maximum-minimum differences (range) suggesting that it produced the most consistent in vivo performance.

No treatment-associated patterns were found in the duration of quantifiable IVM plasma concentrations. Mean IVM concentrations at hr 24 were likewise similar (Table 5).

**Table 5:** Duration of quantifiable IVM plasma concentrations, hr 24 IVM concentrations, and number of dogs exhibiting secondary peaks as a function of treatment

	Number of dogs			ng/mL at hr 24 (mean, %CV)	Number of dogs exhibiting secondary peaks
Treatment	Hr 24	Hr 72	Hr 168		
A	7	20	0	2.30, %CV = 30	16
B	6	21	0	2.45, %CV = 29	13
C	6	20	1	2.59, %CV = 36	12

Many dogs exhibited secondary peaks after one or more of the treatments. In most cases, these secondary peaks did not coincide with a meal, indicating that they were not attributable to enterohepatic circulation (EHC). An example of a dog exhibiting this secondary peak is provided in Supplemental Fig. 2S. The number of dogs exhibiting secondary peaks after each treatment is also provided in Table 5. The number of dogs exhibiting a secondary peak that is considered to be consistent with EHC was 3/12 for Treatment C, 2/13 for Treatment B, and 1/16 for Treatment A.

Plots of the individual dog treatment ratios for AUC0-last, AUC0-3, Cmax and Tmax are in Supplemental Figs. 3S, a, b, c, and d, respectively. An assessment of the minimum and maximum ratios for the various PK parameters and the dogs associated with these ratios are provided in Supplemental Table 2S. Of note is that the number of dogs with between-treatment ratios of A > B, A > C and B > C was similar to that for A < B, A < C and B < C. The fundamental difference is the magnitude of the treatment ratios within the individual subjects.

2. PRZ

**Fig. 5:**
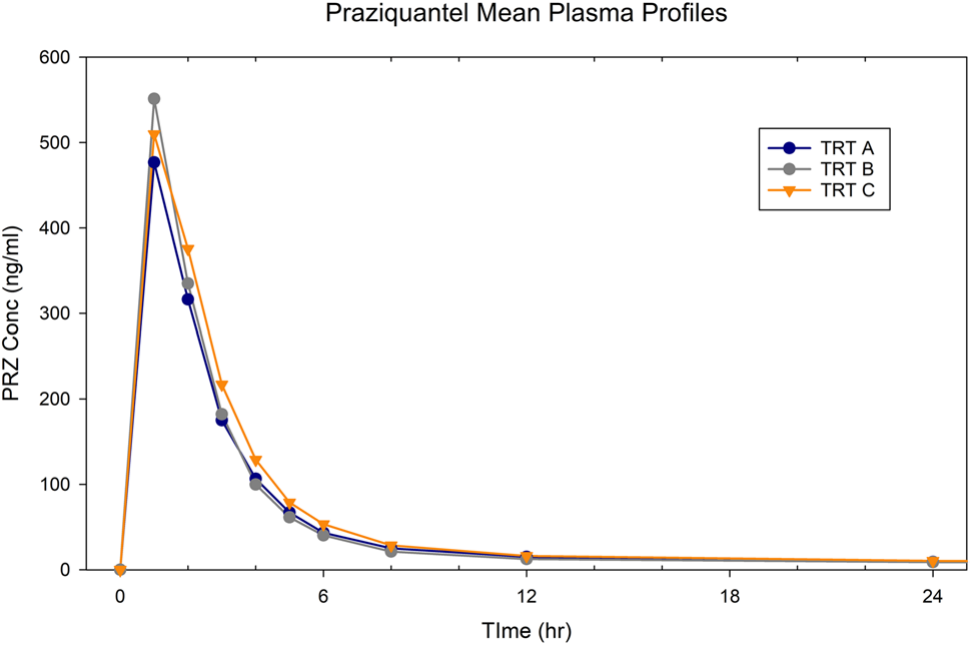
Mean PRZ concentration versus time profile

The average profiles (n = 27 dogs, all treatments) are provided in Fig. 5. The parameter geometric means, minimum, maximum, and range values for each Treatment and the 90% CI for each pairwise comparison is provided in Table 1.

**Table 6:** PRZ confidence intervals and treatment geometric mean values

PRZ	Exponentiated values		Geometric means		Range of values		
Label	Lower CI	Upper CI	Trt	Geomean	Min	Max	Range
AUC0-last (ng×hr/mL)							
A v B	0.71	0.95	A	1029.08	57.45	2541.19	2483.75
B v C	0.81	1.08	B	1250.20	357.82	2722.28	2364.46
A v C	0.67	0.89	C	1332.65	292.57	2974.34	2681.77
Cmax (ng/mL)							
A v B	0.60	0.87	A	387.03	40.37	1382.00	1341.63
B v C	0.88	1.27	B	534.18	182.00	1205.00	1023.00
A v C	0.64	0.92	C	505.54	137.10	1549.00	1411.90
AUC0-2 (ng×hr/mL)							
A v B	0.63	0.91	A	489.70	46.99	1684.26	1637.27
B v C	0.88	1.27	B	645.23	232.32	1454.40	1222.08
A v C	0.67	0.96	C	609.29	167.61	1883.92	1716.32

Based upon the in vitro PRZ dissolution profiles, the expectation was that Treatments A and B would be bioequivalent, but that Treatment C would exhibit a lower Cmax. However, the in vitro dissolution data did not reflect the smaller peak and lower total absorption seen with Treatment A. In fact, Treatment A AUC0-last and Cmax values were significantly less than that of Treatment B, even though both were formulated with PF granules. Treatment C also had significantly higher AUC0-last, Cmax and AUC0-2 values as compared to Treatment A. As seen for IVM, Treatment A had the smallest minimum value across the three PK parameters and Treatment B had the narrowest range. Although Treatments B (PF) and C (PM) were within 0.80–1.25 for AUC0-last, the corresponding 90% CI for Cmax (0.88–1.27) and AUC0-2 (0.88–1.27) suggested that Treatment B exhibited a faster rate of absorption.

Unlike Treatment A, the in vivo relationships between Treatment B and C were consistent with the in vitro dissolution predictions. Therefore, the in vitro dissolution correctly reflected Treatment B vs C relative absorption rates.

Individual dog profiles from hrs 0 – 2 are provided in Supplemental Fig. 4S. Typically, Tmax occurred at 1 h postdose (the first blood sample), indicating a failure to capture the true Tmax. While this underscores the need to have included earlier in vivo sampling times, the shape of the PRZ profiles between hrs 1 vs 1.5 of most dogs make it unlikely that this deficiency markedly biased the conclusions of treatment relative bioavailability.

## Discussion

Challenges associated with the in vivo BE evaluation of locally acting, non-systemically absorbed canine oral formulations prompted the current effort to explore in vitro approaches for assessing product comparability. The possibility of utilizing an in vitro approach has already been explored for other non-systemically absorbed veterinary drug products such as canine cyclosporine ointment [23] and intramammary infusions [24]. For the canine opthalmic cyclosporine ointment and intramammary infusions, a totality of evidence approach was shown to be of value, where not only in vitro release from the formulations but also a characterization of product formulation and critical physicochemical properties were considered important parameters to include for a determination of product BE. Similarly, in the current investigation, we have observed that in vitro dissolution cannot be the sole factor relied upon to confirm product equivalence but rather equivalence assessments need to include considerations with regard to the similarity of product formulations, manufacturing, and their respective dissolution characteristics across a range of pH’s.

It is important to underscore that the purpose of the in vivo BE study reported in this manuscript was solely to utilize these blood level data as “biomarkers” for exploring the ability of comparative in vitro dissolution to describe product relative in vivo dissolution characteristics. The rationale for this approach is that for permeable drugs, a comparison of product in vivo blood levels are influenced primarily by differences in their respective rates and extent of in vivo dissolution and that similarly, a comparative product performance in a clinical endpoint BE trial for locally acting products (where the reference product has already been shown to be safe and effective) is influenced primarily by the comparability of product in vivo dissolution (and any potential excipient effects that can alter drug uptake into the parasite).

Furthermore, despite the experimental nature of this investigation, it would not be appropriate to modify dog physiology (e.g., control of gastric acid secretion) to reduce within and between-dog variability in gastric pH. Since the objective of this investigation was to ascertain the prognostic capability of in vitro dissolution for identifying potential in vivo dissolution inequivalence of non-systemically absorbed drug products when they are administered to an actual patient population, it is necessary to have the animal subjects reflect the range of GI physiological conditions that will be encountered in a normal canine population.

A. Exploring reasons for Treatment A lower than expected oral bioavailability:

1. IVM

As is stipulated in Center for Drug Evaluation and Research guidance titled M9 Biopharmaceutics Classification System (BCS)—Based Biowaivers, multiple media (i.e., three buffer system: pH 1.2, 4.5 and 6.8) are needed to support a determination of in vivo BE in the absence of in vivo BE assessments [25]. In our investigation, the unexpected post-dissolution decline in solubilized IVM under acidic conditions underscores the importance of using a range of pH’s to support an assessment of in vivo BE.

The question is whether the in vitro degradation/precipitation of Treatment A IVM as seen in 0.1 N HCl + 0.2% SLS also occurred in vivo? If it did, did it impact the oral performance of the PRZ (and IVM) components of Treatment A? If faster in vivo dissolution resulted in greater degradation in the stomach of some dogs, it could explain the lower AUC0-last associated with Treatment A as compared to that of B and C. Other investigators also report IVM degradation in 0.1 N HCl after only 1 h of in vitro testing [26]. The pH of the fasted dog stomach can range between pH 1.2 – 6.5 [27]. Although Treatments B and C met the 90% CI BE criteria for AUC0-last, Treatment C had lower AUC0-3 values, an outcome not predicted from the in vitro dissolution profiles.

Another possible source of the observed in vivo/in vitro inconsistencies is that faster release may have resulted in greater exposure to gut P-glycoprotein (P-gp)-associated constraints on the absorption of dissolved drug and therefore greater opportunity to interact with gut Cyp3a12 metabolizing enzymes [28, 29]. In other words, intestinal P-gp may not only limit parent drug absorption by causing an efflux of the drug from the enterocyte (apical membrane) but through repeated cycles of absorption and efflux, can also increase access of drug to metabolism by CYP3A (Cyp3a12 in the dog). Physiologically based pharmacokinetic (PBPK) modeling may help to explore these possibilities.

2. PRZ

In vitro dissolution successfully predicted the PRZ relative bioavailability of Treatment B and C but not that of Treatment A relative to B or C. To explore potential reasons for this outcome, we first considered the within-dog treatment ratios for Cmax, AUC0-last and AUC0-2 (A/B, B/C, A/C) to identify the presence of any discordant observations. Although most dogs had lower exposure after Treatment A vs B or C, there were 11 dogs where AUC0-last was higher after Treatment A than B and 8 dogs with AUC0-last greater after Treatment A than C. Similar relative bioavailability results were associated with Cmax and AUC0-2 (Supplemental Figs. 5a, b, and c, respectively).

We also explored the degree to which the two dogs exhibiting the lowest Treatment A PRZ bioavailability (dog #13, sequence 2, period 3 and dog #24, sequence 3, period 2) influenced the bioinequivalence of Treatments A vs B. Excluding those two dogs from the statistical analysis improved the AUC0-last 90% CI for Treatments A vs B (0.85–1.03) but not early exposure (90% CI for Cmax = 0.71–0.95 and 0.76–1.00 for AUC0-2). Thus, despite the rapid and complete PRZ in vitro release under all test conditions, in vivo equivalence still could not be confirmed. Additional information pertaining to these two dogs, including their corresponding IVM parameter values, is provided in Supplemental Tables 3S and 4S.

**Fig. 6:**
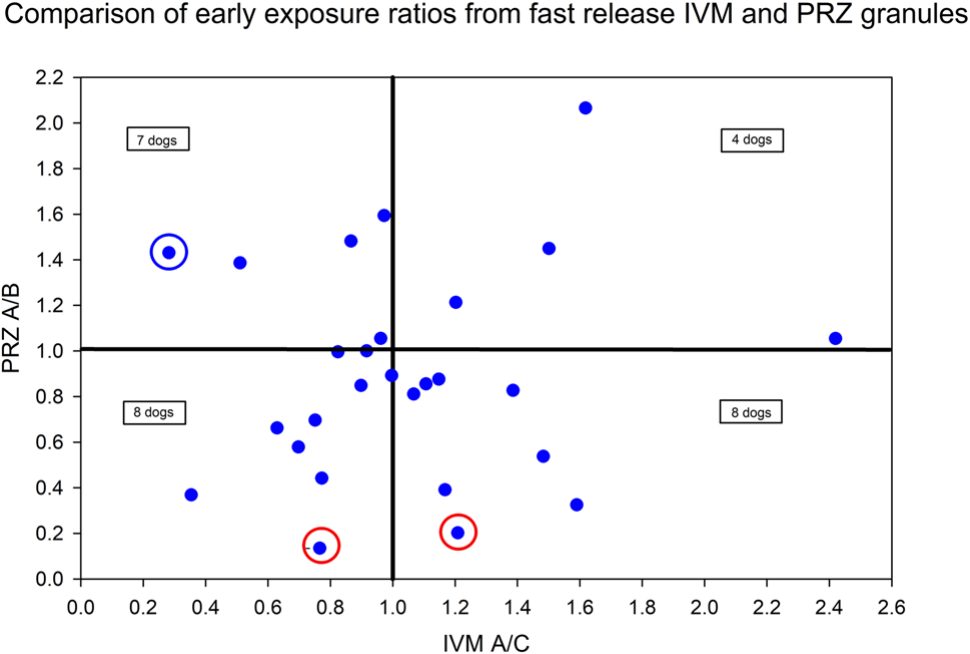
Comparison of early exposure ratios from fast release IVM and PRZ granules

To explore the possibility of a correlation between the IVM and PRZ in vivo failure of Treatment A, the early AUC ratios for fast release IVM (Treatments A/C) was plotted against the early AUC ratios for fast release PRZ granules (Treatments A/B) for each dog. The observed magnitude of dispersion shows no identifiable relationship (Fig. 6).

Dividing the graph into 4 axes, a comparable number of dogs had A < B PRZ but A > C IVM as there were A > B PRZ but A < C IVM. Considering the lower left-hand quadrant, only 1 dog had very low ratios for PRZ and IVM (~ 0.4 for both). The dog with the lowest PRZ ratio (0.13) had an IVM Treatment A/C ratio of 0.77 (circled in red, lower left quadrant) and the dog with a PRZ A/B ratio of 0.2 had an IVM A/C ratio of 1.21 (circled in red, lower right quadrant). However, the dog with the lowest IVM A/C ratio (0.28) had a corresponding A/B PRZ ratio of 1.43 (circled in blue, upper left quadrant). These dogs are illustrative of the lack of consistent differences between Treatment A and the corresponding fast-dissolving granules present in Treatment B (PRZ) or C (IVM), indicating that the failure of Treatment A cannot be ascribed to tablet disintegration failure. Slightly more dogs had a ratio < 0.8 for PRZ (10 dogs) as compared to that for IVM (8 dogs). Therefore, while Treatment A failed to perform in a manner comparable to the corresponding fast release granule for both APIs, the impact of this failure appeared to be more profound for PRZ as compared to IVM.

Also considered was the possibility that a simultaneous release of IVM and PRZ from Treatment A negatively influenced PRZ oral bioavailability since unlike Treatment A, Treatments B and C were formulated to either have a slower release of IVM or of PRZ. However, such interactions were not observed in dogs [30] or people [31], which is consistent with the absence of PRZ influence on transporter activity [32] and the poor or no inhibition by IVM on the human CYP enzymes [33]. Therefore, from a PK perspective, we cannot ascribe an absorption or metabolism-based drug-drug interaction for the lower PRZ bioavailability.

Since PRZ is associated with a high degree of first pass drug loss (as shown in human liver microsomes, where R-PRZ mainly catalyzed by CYP1A1 and 2C19 and S-PRZ mainly by CYP2C19 and CYP3A4) [34]), we cannot exclude the possibility that a higher duodenal and jejunal exposure (canine intestinal segments contains the majority of Cyp3a12) [27] contributed to the lower oral bioavailability associated with Treatment A. Again, PBPK modeling of the in vivo data may provide insights into that possibility.

B. Published examples of in vivo/in vitro relationships with PRZ

In contrast with the results from this investigation, there are numerous examples where in vitro dissolution test results successfully predicted product differences in PRZ oral bioavailability (Table 5). From these published examples, in vitro dissolution has successfully been employed to predict in vivo formulation differences in the rate and extent of PRZ oral bioavailability. Therefore, the reason for the observed failure for Treatment A is still unclear.

**Table 7:** Published examples of in vitro prediction of PRZ in vivo relative bioavailability

Subject	Dosage form	Simulation Conditions	Conclusion	Ref
Fasted Beagle dog	PRZ suspensions, different particle sizes	0.1 N HCL + 0.2% sodium dodecyl sulfate (SDS), paddle 50 rpm	In vitro dissolution test results accurately predicted canine relative bioavailability of PRZ suspensions containing different particle size in 0.1 N HCL + 0.2% SDS but not in a medium of pure water,	Yang et al., 2019 [19]
Rats	PRZ-albumin-bound nanoparticles versus raw PRZ	500 mL volume of ultrapure water maintained at 37 ± 0.5 °C) in a a flow-through type apparatus	The dissolution conditions correctly identified differences in product PRZ oral bioavailability (AUC and Cmax) in rats	Yamasaki et al., 2019 [35]
Fasted Beagle dog	Marketed immediate release tablet versus an experimental extended-release product	0.1 N HCl (paddle, 100 rpm, no surfactant)	Product differences in PRZ rate of in vivo release (as estimated by mean residence time and Tmax) was accurately predicted by the in vitro dissolution test	Wen et al., 2021 [17]
Healthy human male volunteers	Six brands of film coated PRZ tablets	Simulated gastric fluids without enzymes (pH 1.2) and simulated intestinal fluid without enzyme (pH 7.5)	The ability to identify inequivalence across brands of PRZ tablets was greater when in vitro tests were conducted in acidic rather than more basic conditions	Kaojarern et al., 1989 [36]

C. Additional perspectives on the use of in vitro dissolution to compare formulations for locally acting GI drugs:

Han et al., identified challenges associated with use of either systemic blood level BE or in vitro dissolution when comparing formulations of the locally acting compound, budesonide, in humans [37]. They showed that while in vitro dissolution can be predictive of in vivo drug exposure in the lower portions of the small intestine and in the colon, the upper portion of the small intestine is much more sensitive to formulation differences. Based upon their PBPK investigation, they suggested that if the upper portion of the GI tract is being targeted for a conclusion of product equivalence, the f2 value defining comparability should be increased to 65 (healthy humans). An even higher f2 value (f2 = 75) was suggested for Crohn’s disease patients (exposure within the ileum). Their conclusions underscore the importance of understanding the targeted gut location and the drug/drug product physicochemical characteristics.

## Conclusions

Efforts were made to seek potential reasons for the unexpected in vivo/in vitro relationships identified in this investigation. However, although the potential impact for any of these possibilities were not identified, the study results succeeded in demonstrating the need to tightly control the extent to which products could differ and remain eligible for the application of an in vitro BE approach. Importantly, the results of this investigation underscored the need to consider more than in vitro dissolution data alone when evaluating the BE of locally acting veterinary oral products. Rather, a totality of evidence approach was shown to be necessary. Points that would need to be considered include product comparative in vitro dissolution across a range of conditions (e.g., as was needed for identifying the unanticipated degradation of IVM in Treatments A and C when tested in the 0.1 N HCL + 0.2% SLS), an understanding of the GI tract location where the product exerts its therapeutic effects (i.e., how critical is comparability in rates of in vivo dissolution), and a comparison of drug product composition and manufacturing method (e.g., not only formulation but also granule sizes differed across the three treatments). Applying such an approach would have alerted us to potential inequivalence across the three treatments used in this study.

Ultimately, adopting a totality of evidence approach will enable the need for clinical endpoint BE trials to be limited to situations where in vitro BE assessments are not applicable or when BE cannot be concluded when using an in vitro approach, thereby achieving our goal to reduce, refine, and replace the need for animal studies.

## Supplementary Information

Supplementary Material 1.

## Data Availability

The in vivo and in vitro datasets are available upon request.

## References

[1] Charmot D. Non-systemic drugs: a critical review. Curr Pharm Des. 2012;18(10):1434–45. 10.2174/138161212799504858. 22300258 10.2174/138161212799504858 PMC3343347

[2] FDA Guidance for industry. Guidance #35 Bioequivalence, November 2006. https://www.fda.gov/regulatory-information/search-fda-guidance-documents/cvm-gfi-35-bioequivalence-guidance Accessed 06/04/2024.

[3] Bermingham E, Del Castillo JR, Lainesse C, Pasloske K, Radecki S. Demonstrating bioequivalence using clinical endpoint studies. J Vet Pharmacol Ther. 2012;35(Suppl 1):31–7. 10.1111/j.1365-2885.2012.01366.x. 22413789 10.1111/j.1365-2885.2012.01366.x

[4] Animal Use Alternatives (3Rs). USDA https://www.nal.usda.gov/animal-health-and-welfare/animal-use-alternatives Accessed 06/04/2024.

[5] What is antiparasitic resistance. https://www.fda.gov/animal-veterinary/safety-health/antiparasitic-resistance Accessed 06/04/2024.

[6] Martinez MN, Fahmy R. Demonstrating comparative in vitro bioequivalence for animal drug products through chemistry and manufacturing controls and physicochemical characterization: a proposal. AAPS J. 2015;17(2):307–12. 10.1208/s12248-014-9702-8. 25609223 10.1208/s12248-014-9702-8 PMC4365090

[7] Amidon GL, Lennernäs H, Shah VP, Crison JR. A theoretical basis for a biopharmaceutic drug classification: the correlation of in vitro drug product dissolution and in vivo bioavailability. Pharm Res. 1995;12(3):413–20. 10.1023/a:1016212804288. 7617530 10.1023/a:1016212804288

[8] NADA 141–441, IVERHART MAX® Chew (ivermectin/pyrantel pamoate/praziquantel) (virbac.com) https://us.virbac.com/iverhart-max-chew. Accessed 06/04/2024.

[9] Hollenbeck RG, Fahmy R, Martinez MN, Ibrahim A, Hoag SW. Design and process considerations for preparation of modified release ivermectin granules and praziquantel granules by wet granulation. Submitted for publication, 10.1208/s12249-024-03030-2 39843849

[10] Revision USP Bulletin dated 2/01/2008, USP 29, ivermectin tablets, https://www.uspnf.com/sites/default/files/usp_pdf/EN/USPNF/ivermectinTablets.pdf Accessed 07/18/2024.

[11] USP 29, Praziquantel tablets, http://www.pharmacopeia.cn/v29240/usp29nf24s0_m68458.html Accessed 07/18/2024.

[12] Ibrahim A, Wang F, Hollenbeck RG, Martinez MN, Fahmy R, Hoag SW (2023) Development and validation of a stability-indicating UPLC-DAD method for the simultaneous determination of ivermectin and praziquantel in pharmaceutical tablets and dissolution media. AAPS PharmSciTech. 2023;24(7):211. 10.1208/s12249-023-02656-y. 10.1208/s12249-023-02656-y 37821763

[13] Martin-Pastor M, Stoyanov E. New insights into the use of hydroxypropyl cellulose for drug solubility enhancement: An analytical study of sub-molecular interactions with fenofibrate in solid state and aqueous solutions. J Polym Sci. 2021;59(16):1855–65. 10.1002/pol.20210240.

[14] Yamada T, Saito N, Imai T, Otagiri M. Effect of grinding with hydroxypropyl cellulose on the dissolution and particle size of a poorly water-soluble drug. Chem Pharm Bull (Tokyo). 1999;47(9):1311–3. 10.1248/cpb.47.1311. 10517010 10.1248/cpb.47.1311

[15] Sugita M, Kataoka M, Sugihara M, Takeuchi S, Yamashita S. Effect of excipients on the particle size of precipitated pioglitazone in the gastrointestinal tract: impact on bioequivalence. AAPS J. 2014;16(5):1119–27. 10.1208/s12248-014-9646-z. 25070482 10.1208/s12248-014-9646-z PMC4147060

[16] Al-Azzam SI, Fleckenstein L, Cheng KJ, Dzimianski MT, McCall JW. Comparison of the pharmacokinetics of moxidectin and ivermectin after oral administration to beagle dogs. Biopharm Drug Dispos. 2007;28(8):431–8. 10.1002/bdd.572. 17847063 10.1002/bdd.572

[17] Wen X, Deng Z, Xu Y, Yan G, Deng X, Wu L, Liang Q, Fang F, Feng X, Yu M, He J. Preparation and in vitro/in vivo evaluation of orally disintegrating/modified-release praziquantel tablets. Pharmaceutics. 2021;13(10): 1567. 10.3390/pharmaceutics13101567. 38324. 10.3390/pharmaceutics13101567 PMC8538324 34683860

[18] Liu Y, Wang T, Ding W, Dong C, Wang X, Chen J, Li Y. Dissolution and oral bioavailability enhancement of praziquantel by solid dispersions. Drug Deliv Transl Res. 2018;8(3):580–90. 10.1007/s13346-018-0487-7. 29450806 10.1007/s13346-018-0487-7

[19] Yang R, Zhang T, Yu J, Liu Y, Wang Y, He Z. Asian J Pharm Sci. 2019;14(3):321–8. 10.1016/j.ajps.2018.06.001. 32104462 10.1016/j.ajps.2018.06.001 PMC7032129

[20] Steiner K, Garbe A, Diekmann HW, Nowak H. The fate of praziquantel in the organism I Pharmacokinetics in animals. Eur J Drug Metab Pharmacokinet. 1976;1:85–95. 10.1007/BF03189262.

[21] FDA CDER Guidance for Industry:M13A Bioequivalence for Immediate-Release Solid Oral Dosage Forms. JANUARY 2023. https://www.fda.gov/regulatory-information/search-fda-guidance-documents/m13a-bioequivalence-immediate-release-solid-oral-dosage-forms Accessed 06/04/2024.

[22] FDA CDER Guidance for Industry, August 1997: Dissolution Testing of Immediate Release Solid Oral Dosage Forms https://www.fda.gov/regulatory-information/search-fda-guidance-documents/dissolution-testing-immediate-release-solid-oral-dosage-forms Accessed 06/04/2024.

[23] Dong Y, Qu H, Pavurala N, Wang J, Sekar V, Martinez MN, Fahmy R, Ashraf M, Cruz CN, Xu X. Formulation characteristics and in vitro release testing of cyclosporine ophthalmic ointments. Int J Pharm. 2018;544(1):254–64. 10.1016/j.ijpharm.2018.04.042. 29684560 10.1016/j.ijpharm.2018.04.042

[24] Helal NA, Martinez MN, Longstaff DG, Rahman Z, Nutan MTH, Khan MA. Development and validation of Matrix of Chemistry, Manufacturing, and Control (MoCMC) system for intramammary drug products (IMM). Pharm Res. 2024;41(5):1007–20. 10.1007/s11095-024-03689-z. 38561579 10.1007/s11095-024-03689-z

[25] FDA CDER, CBER Guidance for Industry: M9 Biopharmaceutics Classification System-Based Biowaivers Guidance for Industry https://www.fda.gov/media/148472/download Accessed 07/18/2024

[26] Sznitowska M, Pietkiewicz J, Stokrocka M, Janicki S. Dissolution test for ivermectin in oral veterinary paste. Pharmazie. 2004;59(10):814–5 PMID: 15544065. 15544065

[27] Martinez MN, Mochel JP, Neuhoff S, Pade D. Comparison of canine and human physiological factors: understanding interspecies differences that impact drug pharmacokinetics. AAPS J. 2021;23(3):59. 10.1208/s12248-021-00590-0. 33907906 10.1208/s12248-021-00590-0

[28] Cummins CL, Jacobsen W, Benet LZ. Unmasking the dynamic interplay between intestinal P-glycoprotein and CYP3A4. J Pharmacol Exp Ther. 2002;300(3):1036–45. 10.1124/jpet.300.3.1036. 11861813 10.1124/jpet.300.3.1036

[29] Benet LZ, Cummins CL. The drug efflux-metabolism alliance: biochemical aspects. Adv Drug Deliv Rev. 2001;50(Suppl 1):S3-11. 10.1016/s0169-409x(01)00178-8. 11576692 10.1016/s0169-409x(01)00178-8

[30] Ozdemir Z, Faki HE, Uney K, Tras B. Investigation of pharmacokinetic interaction between ivermectin and praziquantel after oral administration in healthy dogs. J Vet Pharmacol Ther. 2019;42(5):497–504. 10.1111/jvp.12769. 31183888 10.1111/jvp.12769

[31] Na-Bangchang K, Kietinun S, Pawa KK, Hanpitakpong W, Na-Bangchang C, Lazdins J. Assessments of pharmacokinetic drug interactions and tolerability of albendazole, praziquantel and ivermectin combinations. Trans R Soc Trop Med Hyg. 2006;100(4):335–45. 10.1016/j.trstmh.2005.05.017. 16271272 10.1016/j.trstmh.2005.05.017

[32] Kigen G, Edwards G. Drug-transporter mediated interactions between anthelminthic and antiretroviral drugs across the Caco-2 cell monolayers. BMC Pharmacol Toxicol. 2017;18(1):20. 10.1186/s40360-017-0129-6. 28468637 10.1186/s40360-017-0129-6 PMC5415745

[33] Rendic SP. Metabolism and interactions of ivermectin with human cytochrome P450 enzymes and drug transporters, possible adverse and toxic effects. Arch Toxicol. 2021;95(5):1535–46. 10.1007/s00204-021-03025-z. 33719007 10.1007/s00204-021-03025-z PMC7956433

[34] Kapungu NN, Li X, Nhachi C, Masimirembwa C, Thelingwani RS. In vitro and in vivo human metabolism and pharmacokinetics of S- and R-praziquantel. Pharmacol Res Perspect. 2020;8(4):e00618. 10.1002/prp2.618. 32700798 10.1002/prp2.618 PMC7376644

[35] Yamasaki K, Taguchi K, Nishi K, Otagiri M, Seo H. Enhanced dissolution and oral bioavailability of praziquantel by emulsification with human serum albumin followed by spray drying. Eur J Pharm Sci. 2019;139:105064. 10.1016/j.ejps.2019.105064. 31491499 10.1016/j.ejps.2019.105064

[36] Kaojarern S, Nathakarnkikool S, Suvanakoot U. Comparative bioavailability of praziquantel tablets. DICP. 1989;23(1):29–32. 10.1177/106002808902300105. 2718480 10.1177/106002808902300105

[37] Han C, Sun T, Chirumamilla SK, Bois FY, Xu M, Rostami-Hodjegan A. Understanding Discordance between In Vitro Dissolution, Local gut and systemic bioequivalence of budesonide in healthy and Crohn’s Disease patients through PBPK modeling. Pharmaceutics. 2023;15(9):2237. 10.3390/pharmaceutics15092237. 37765205 10.3390/pharmaceutics15092237 PMC10535222

