# Supplementary material for: The Use of Systemically Absorbed Drugs to Explore An In Vitro Bioequivalence Approach For Comparing Non-Systemically Absorbed Active Pharmaceutical Ingredients in Drug Products For Use in Dogs
Source: Pharm Res. 2024 Sep 9;41(9):1797–809. doi: 10.1007/s11095-024-03766-3 (PMC11436403; doi:10.1007/s11095-024-03766-3)
Supplement: Supplementary file 1 — Supplementary Material 1. [file 11095_2024_3766_MOESM1_ESM.docx]

**SUPPLEMENTAL INFORMATION:**

Table of Contents:

1. Figures

Figure 1Sa, b: IVM chromatograms showing degradation in 0.1 N HCl. Page 2-3

Figure 2S: Example of a dog exhibiting a secondary peak after receiving Treatment A. Page 3

Figure 3Sa, b, c, d: Within dog pairwise comparison of IVM PK parameters Page 4-5

Figure 4S: Individual dog early PRZ concentration versus time profiles. Page 6

a: Treatment A; b: Treatment B; c: Treatment C

Figure 5S: Within dog PRZ treatment ratios for a) AUC0-last; b) Cmax; and c) AUC0-2 Page 7-8

1. Tables

Table 1S: Results of the in vitro dissolution study Page 9

Table 2S: Dogs with minimum and maximum IVM ratios for each set of treatment Page 10 values for Cmax, AUC0-last and AUC0-3.

Table 3S: Evaluation of IVM and PRZ between treatment ratios for dogs #13 and 24 Page 10

Table 4S: Mean IVM and PRZ value dog dogs 13 and 24 relative to the corresponding Page 11

geometric mean for that parameter and Tablet formulation

1. Drug Pharmacokinetic Information Page 11-12
2. Study Design: Page 12
3. In Vivo Plasma Sample Analytical Methods Page 13-14
4. Solubility Test Considerations: Page 14
5. References Page 14-15

**
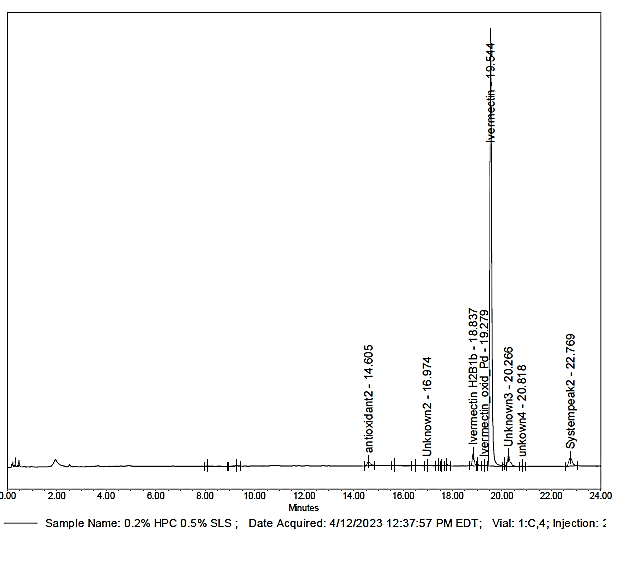
Figure 1Sa:** IVM chromatograms obtained from the “Water + 0.2% HPC + 0.5% SLS” solvent system. In this case, the IVM peak is clearly evident and there are minimal peaks associated with the unknown substances.


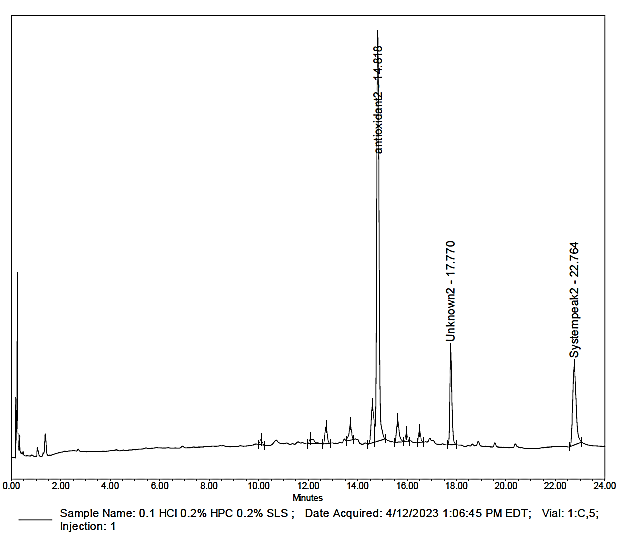
**Figure 1Sb**: IVM chromatograms after exposure to 0.1N HCl + 0.2% HPC + 0.2% SLS. In this case, there is a large downstream peak that is most likely ivermectin degradation product that obscures the IVM peak.

**Figure 2S: Example of a dog exhibiting a secondary IVM peak after Treatment A.**

The parameter ratios for the individual dogs were plotted to explore the possibility of a few dogs that had discordant results (Supplemental Figures 3a, b and c). A wide scatter is observed in these plots, indicating that discordant results could not be identified and that the confidence intervals reflect the trend in relative bioavailability across all comparisons. The corresponding minimum and maximum ratios and the dogs associated with these ratios are provided in Supplemental Table 1.

**Figure 3S.** Figures a, b, and c illustrate the inter-subject scatter in the treatment ratios, with those involving treatment C appearing to exceed that of A/B for AUC0-last but with similar variability about the three treatment ratios for AUC0-3, and Cmax. Figure d provides the individual Tmax values as a function of treatment.

**Figure 3Sa: Between dog comparison of IVM AUC0-last treatment ratios**

**Figure S3b: Between dog comparison of IVM AUC0-3 treatment ratios**

**Figure3Sc: Between dog comparison of IVM Cmax treatment ratios**

**Figure 3Sd: IVM Tmax values for the individual dogs as a function of treatment**

**Figure 4S: Individual dog early PRZ concentration versus time profiles as a function of treatment**

**Figure 4Sa: Treatment A**

**Figure 4Sb**

**Figure 4Sc**

**Figure 5S: Inter-subject scatter in the treatment ratios for PRZ.**

**Figure 5Sa: Between dog comparison of PRZ AUC0-last treatment ratios**

**Figure 5Sb: Between dog comparison of PRZ AUC0-2 treatment ratios**

**Figure 5Sc: Between dog comparison of PRZ Cmax treatment ratios**

**Table 1S: In vitro dissolution results under each set of conditions. Results are expressed as mean and %CV at each timepoint for IVM and for PRZ**


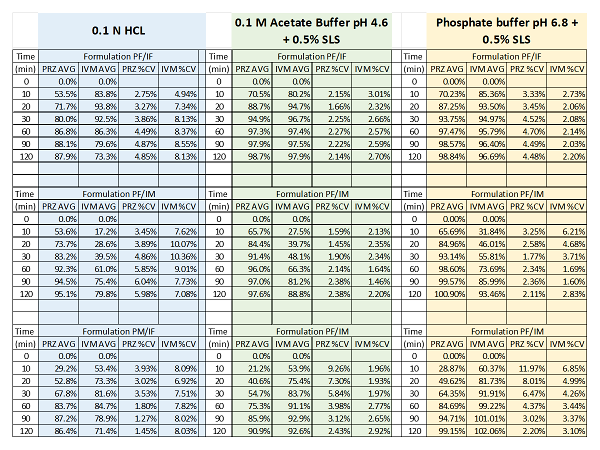


**Table 2S: Dogs with minimum and maximum IVM ratios for each set of treatment values for Cmax, AUC0-last and AUC0-3.**

|  | Cmax | | |  | AUC0-last | | |  | AUC0-3 | | | |
| --- | --- | --- | --- | --- | --- | --- | --- | --- | --- | --- | --- | --- |
|  | A/B | A/C | B/C |  | A/B | A/C | B/C |  | A/B | A/C | B/C |  |
| Min | 0.10 | 0.15 | 0.50 |  | 0.16 | 0.09 | 0.41 |  | 0.10 | 0.28 | 0.64 |  |
| Max | 1.88 | 1.84 | 2.91 |  | 1.95 | 3.48 | 3.20 |  | 1.82 | 2.42 | 4.16 |  |
| Min | dog 6 | dog 6 | dog 9 |  | dog 6 | dog 6 | dog 21 |  | dog 6 | dog 6 | dog 9 |  |
| Max | dog 18 | dog 23 | dog 22 |  | dog 10 | dog 23 | dog 3 |  | dog 11 | dog 22 | dog 22 |  |
| # dogs >1 | 14 | 13 | 13 |  | 13 | 14 | 13 |  | 9 | 12 | 16 |  |

**Table 3S: Evaluation of IVM and PRZ between treatment ratios for dogs #13 and 24**

| IVM | | | | |
| --- | --- | --- | --- | --- |
| Trt | Dog | Cmax (ng/mL) | AUC0-last (ng*hr/mL) | AUC0-3 (ng*hr/mL) |
| A/B | 13 | 1.13 | 1.27 | 1.16 |
| A/B | 24 | 0.99 | 1.41 | 0.92 |
| A/C | 13 | 0.78 | 0.79 | 0.77 |
| A/C | 24 | 1.28 | 1.11 | 1.21 |
| B/C | 13 | 0.69 | 0.62 | 0.66 |
| B/C | 24 | 1.30 | 0.78 | 1.31 |
| PRZ | | | | |
| Trt | Dog | Cmax (ng/mL) | AUC0-last (ng*hr/mL) | AUC0-2 (ng*hr/mL) |
| A/B | 13 | 0.14 | 0.21 | 0.13 |
| A/B | 24 | 0.22 | 0.16 | 0.20 |
| A/C | 13 | 0.07 | 0.13 | 0.08 |
| A/C | 24 | 0.29 | 0.20 | 0.28 |
| B/C | 13 | 0.51 | 0.62 | 0.57 |
| B/C | 24 | 1.33 | 1.22 | 1.39 |

The corresponding parameter values relative to the geometric means for each treatment are provided in Table 4S.

**Table 4S: Mean IVM and PRZ value for dogs 13 and 24 relative to the corresponding geometric mean for that parameter and treatment (red reflects highly discordant values)**

| **IVM** | | | **PRZ** | | |  |
| --- | --- | --- | --- | --- | --- | --- |
| **Tablet** | **A** | **B** | **C** | **A** | **B** | **C** |
| Dog # | Cmax | | | | | |
| 13 | 12.97 | 11.44 | 16.64 | **47.67** | 336.30 | 656.90 |
| 24 | 8.67 | 8.77 | 6.75 | **40.37** | 182.00 | 137.10 |
| Geom mean | 8.14 | 9.37 | 8.79 | 387.03 | 534.18 | 505.54 |
|  | AUC0-last | | | | | |
| 13 | 219.27 | 172.78 | 277.08 | **183.21** | 878.54 | 1406.70 |
| 24 | 200.80 | 142.04 | 181.27 | **57.45** | 357.82 | 292.57 |
| Geom mean | 137.60 | 153.89 | 156.43 | 1029.08 | 1250.20 | 1332.65 |
|  | Early AUC | | | | | |
| 13 | 30.67 | 26.56 | 40.04 | **56.95** | 422.54 | 744.58 |
| 24 | 18.13 | 19.68 | 15.01 | **46.99** | 232.32 | 167.61 |
| Geom mean | 15.10 | 18.99 | 15.92 | 489.70 | 645.23 | 609.29 |

**C. Drug Information:**

IVM: a semi-synthetic product produced by the fermentation of avermectins B1 and B2. It is a mixture containing about 90% ivermectin B1a (5-O-demethyl-22,23-ihydroavermectin A1a; also referred to as component H2B1a) and 10% ivermectin B1b (5-O-demethyl-25-de(1-methylpropyl)-25-(1-methylethyl)-22,23-dihydroavermectin A1a; also referred to as component H2B1b) [1]. Although highly permeable (e.g., considered a BCS II compound in humans) [2], its rate and extent of oral absorption (as demonstrated in human subjects) are related to product formulation [3]. IVM is a large highly lipophilic molecule which is insoluble in water (0.0040 mg drug dissolved/mL water). It is expected to have good passive membrane permeability (based upon the calculated log of the partitioning of the drug between octanol and water, Log Pow of approximately 3.22 [4]. The World Health Organization (WHO) Lists it as a Biopharmaceutics Classification System (BCS) Class 2/4 drug [5]. This is consistent with its experimental extrinsic membrane permeability [6]. IVM systemic exposure is influenced by CYP3A-mediated metabolism and P-gp-mediated efflux, with peak concentrations occurring at about 4 hrs after the administration of tablets in fasted human subjects [2]. IVM is neutral at all pH’s [7]. IVM appears to undergo substantial intestinal secretion (which could lead to drug reabsorption) and given the concentrations observed in bile, is likely to potentially exhibit enterohepatic circulation (EHC). Either of these two phenomena could result in an increase in estimated drug exposure (area under the concentration versus time curve, AUC) [8].

PRZ: an isoquinolin-4-one that contains an asymmetric center in position 11b. The commercial preparation is a racemate composed of equal parts of ‘‘levo’’ R(–) and ‘‘dextro’’ S(+) isomers. Only the (–)-enantiomer is endowed with antischistosomal activity [9]. Its serum protein binding is 71% in dogs and elimination of the parent compound is exclusively by metabolism [10].

PRZ is associated with low aqueous solubility but high passive permeability [11, 12]. The WHO classifies it as a BCS Class 2 compound [5]. Its low oral bioavailability has been attributed to a high first pass effect [13]. In humans, praziquantel is metabolized by numerous CYPs, including CYP3A4 [14]. The canine orthologue, Cyp3a12, is located in the dog upper and lower jejunum and duodenum, with abundance and activity declining from small intestine to the colon [15]. Therefore, we need to consider the possibility that rapid release into the upper portion of the small intestine may lead to greater metabolism in the gut than would occur if the release of drug was delayed until further down the GI tract.

PRZ is not a P-gp substrate [16]. However, Hayeshi et al., 2006 suggested that the possibility of active transport cannot be excluded because while little difference in direction was seen at 20 µM (= 0.00625 mg/mL and where ratio of means of A to B/B to A = 1.14), it increased to 1.25 at 100 µM and to 1.45 at 200 µM (which equals 0.0624822 mg/mL for a praziquantel MW of 312.411) [1]. Nevertheless, based upon their estimated ratios, even if some active transport was involved in absorption process, it would be a very small of the overall absorption process and therefore not likely to influence our study results.

**D. Study Design:**

The design of the study was as follows:

Sequence 1: Treatment A (Period 1), Treatment B (Period 2), Treatment C (Period 3)

Sequence 2: Treatment B (Period 1), Treatment C (Period 2), Treatment A (Period 3)

Sequence 3: Treatment C (Period 1), Treatment A (Period 2), Treatment B (Period 3)

**Dog information**: Intact male Beagles (*Canis familiaris*), were employed. The dogs were obtained from a USDA certified breeder of laboratory Beagles. While at the vendor facility, all dogs were vaccinated against *Bordetella bronchiseptica*, Parvovirus Distemper Leptospira, Rabies, and Papilloma virus (Types 1 and 2). At 3.5 – 18 weeks of age, they were administered amprolium and sulfadiamethoxine to prevent coccidia infection. From 3.5 – 8 weeks, they were administered pyrantel pamoate (5 mg/kg) every two weeks and then monthly thereafter. Lastly, at the vendor facility, they were also administered IVM (0.009 mg/kg) in their feed. A health certificate for all animals was obtained from the vendor at the time of animal delivery, along with relevant USDA transportation documentation.

Upon arrival at OAS, they were placed in quarantine for 14 days and given an initial health assessment including: vital parameters, oral exam, thoracic auscultation, abdominal palpation, weight measurement, and testing for heartworm and Lyme’s disease. The dogs were pair-housed (except for one set of triple housed dogs) for approximately three months prior to the first drug administration. Following the final dose, the dogs were neutered and held in quarantine for two weeks to ensure their health and readiness to be retired for adoption. All 27 dogs completed the study and were adopted as family pets. All dogs were given a 3-year rabies vaccination prior to the neuter surgeries.

**E. In Vivo Analytical Method:**

Beagle dog plasma samples were analyzed using the following LC-MS/MS procedure:

Whole blood samples were maintained on wet ice and subsequently transferred to the analytical laboratory at the CVM OAS for dog plasma generation on the same day as sample collection. A cooler filled with wet ice and a facility vehicle were used for sample transportation between animal research facility and the analytical lab. The dog plasma samples were generated by centrifuging the whole blood sample tube at 3000 rpm (x 1700 g), at 2 to 8^o^C, for 10 min. The plasma was then transferred to a polypropylene centrifuge tube and stored at < -70^o^C prior to LC-MS/MS analysis.

The LC-MS/MS method, which was initially developed and validated under non-GLP study, was qualified with modification for GLP study use. All the study samples were analyzed under this GLP qualified LC-MS/MS method. Briefly, an aliquot of 100 µL plasma sample was added with 25 µL diluted working internal standard. After adding 400 µL of acetonitrile as a protein precipitation solvent, all samples were vortexed well and centrifuged. The supernatants were transferred for direct injection on LC-MS/MS system.

Inter-assay precision and accuracy was calculated from the average of three validation batches. Results showed average % CV for the lowest QC 0 (LLOQ) as 7.34% for IVM, and 11.0% for PRZ. All other QCs as ≤ 7.08% for IVM, and ≤ 5.24% for PRZ. Average percentage difference from the theoretical concentration for the lowest QC 0 (LLOQ) was 1.92% for IVM and 1.24% for PRZ. All other QCs was ≤ 7.71% for IVM and ≤ 4.41% for PRZ. All the inter-assay results met the performance criteria for average %CV and percentage difference from the theoretical concentration (accuracy) ≤ 20% for LLOQ and ≤ 15% for all other QCs specified in the FDA “Bioanalytical Method Validation Guideline for Industry”.

The LC-MS/MS system was a Shimadzu LC-30AD series liquid chromatography coupled with an AB Sciex 5500 QTRAP mass spectrometer. The Shimadzu LC-30AD series liquid chromatography was composed of a degasser, a solvent delivery module, and an auto-sampler, a column oven, which were controlled by Shimadzu controller. A Waters UPLC column, HSS T3 (2.1 x 50 mm, 1.8 µm) with a VanGuard pre-column (2.1 x 5 mm, 1.8 µm) was used for the chromatographic separation and the column oven temperature kept at 35^o^C. The mobile phase A consisted of water with 0.1% formic acid and 5 mM ammonium formate, and mobile phase B consisted of methanol with 0.1% formic acid and 5 mM ammonium formate. The total flow rate was set at 0.40 mL/min with a gradient program. The total run time for each injection was 11 min with divert valve switching to waste after both analytes eluted and a 10 µL injection volume.

The AB Sciex 5500 QTRAP mass spectrometer was installed with an electrospray ionization (ESI) source and integrated with a divert valve for switching the LC flow between waste line and mass spectrometer.  The ESI source was operated under positive mode with multiple reaction monitoring (MRM) with ion transition of m/z 313.3 $\to$ 174.1 for PRZ, m/z 324.4 $\to$204.1 for PRZ-d11 (internal standard), m/z 892.6 $\to$ 569.4 for IVM, and m/z 890.7 $\to$ 305.3 for ABM (internal standard).  Both PRZ and PRZ-d11 eluted at approximately 1.4 min, while as IVM eluted at approximate 2.62 min, and ABM eluted at approximate 2.32 min, respectively.

The duplicate matrix-matched standard curves, run acceptance quality controls at low, mid, and high levels, minimum three replicate of three at each level for both IVM and PRZ were the key components for a typical analytical sample analysis batch. Up to x 14 time-point study samples collected from one study subject were analyzed within one sample extraction batch. Study sample results could only be reported under the conditions of meeting the run acceptance criteria of this sample analysis batch. Any study samples analyzed within a failed sample analysis batch were required to be re-analyzed. For any study sample with no data or no spectrum acquired during injection caused by system software error, it was reported either as N/A (not available) or re-analyzed if enough aliquot sample volume available for re-extraction. For any questionable data, if the samples were re-analyzed, the re-analyzed data averaged from duplicate measurement were reported, and rejected the previous questionable data. For the randomly selected samples for incurred sample re-assay (ISR) to demonstrate assay repeatability and data reliability, the original results acquired from their first measurement were reported.

**F. Solubility Test Considerations:**

Due to solubility constraints, only PRZ was tested in buffer alone (i.e., without the inclusion of HPC or SLS). The test tubes were shaken for 24 hrs at 37^0^ C. Samples were withdrawn, filtered through depth filter to remove excess powder and immediately diluted 1:1 to prevent temperature induced precipitation. The ultra-performance liquid chromatography (UPLC) samples were prepared as a 1:50 dilution and filtered through a syringe filter. When the IVM peak was too small to be detected, the undiluted sample was injected. For PRZ, the UPLC samples were prepared as 1:10 dilution and filtered through a syringe filter (some samples with PRZ + SLS were cloudy).

**G. References:**

1. Drugbank online: Ivermectin. <https://go.drugbank.com/drugs/DB00602> Accessed 06/04/24).

2. Rowland Yeo K, Wesche D. PBPK modeling of ivermectin-Considerations for the purpose of developing alternative routes to optimize its safety profile. CPT Pharmacometrics Syst Pharmacol. 2023; 12(5):598-609. https://doi: 10.1002/psp4.12950.

3. Ceballos L, Alvarez L, Lifschitz A, Lanusse C. Ivermectin systemic availability in adult volunteers treated with different oral pharmaceutical formulations. Biomed Pharmacother. 2023; 160:114391. https://doi: 10.1016/j.biopha.2023.114391.

4. Edwards CA, Atiyeh RM, Rombke J. Environmental impact of avermectins. Rev Environ Contam Toxicol. 2001:171:111–37. https:// doi:10.1007/978-1-4613-0161-5_3.

5. World Health Organization (WHO) Technical Report Series, No. 937, 2006 Annex 8, Proposal to waive in vivo bioequivalence requirements for WHO Model List of Essential Medicines immediate-release, solid oral dosage forms.

6. Escher BI, Berger C, Bramaz N, Kwon JH, Richter M, Tsinman O, Avdeef A. Membrane-water partitioning, membrane permeability, and baseline toxicity of the parasiticides ivermectin, albendazole, and morantel. Environ Toxicol Chem. 2008; 27(4):909-18. https://doi: 10.1897/07-427.1.

7. Liebig M, Fernandez AA, Blübaum-Gronau E, Boxall A, Brinke M, Carbonell G, Egeler P, Fenner K, Fernandez C, Fink G, Garric J, Halling-Sørensen B, Knacker T, Krogh KA, Küster A, Löffler D, Cots MA, Pope L, Prasse C, Römbke J, Rönnefahrt I, Schneider MK, Schweitzer N, Tarazona JV, Ternes TA, Traunspurger W, Wehrhan A, Duis K. Environmental risk assessment of ivermectin: A case study. Integr Environ Assess Manag. 2010;6 Suppl:567-87. https://doi: 10.1002/ieam.96. Erratum in: Integr Environ Assess Manag. 2010; 6(4):790. PMID: 20821718.

8. Laffont CM, Toutain PL, Alvinerie M, Bousquet-Mélou A. Intestinal secretion is a major route for parent ivermectin elimination in the rat. Drug Metab Dispos. 2002; 30(6):626-30. https://doi: 10.1124/dmd.30.6.626.

9. Cioli D, Pica-Mattoccia L. Praziquantel. Parasitol Res. 2003; 90 Supp 1:S3-9. https://doi: 10.1007/s00436-002-0751-z.

10. Committee for Veterinary Medicinal Products, Praziquantel Summary Report, EMEA/MRL/141/96-FINAL,1996. <https://www.ema.europa.eu/en/documents/mrl-report/praziquantel-summary-report-1-committee-veterinary-medicinal-products_en.pdf>. Accessed 6/04/24.

11. Eason T, Ramirez G, Clulow AJ, Salim M, Boyd BJ. Revisiting the dissolution of praziquantel in biorelevant media and the impact of digestion of milk on drug dissolution. Pharmaceutics. 2022; 14(10):2228. https://doi.org/10.3390/pharmaceutics14102228

12. González-Esquivel D, Rivera J, Castro N, Yepez-Mulia L, Jung Cook H. In vitro characterization of some biopharmaceutical properties of praziquantel. Int J Pharm. 2005; 13;295(1-2):93-9. https://doi: 10.1016/j.ijpharm.2005.01.033.

## 13. Gandhi RG and Elshaboury RH. Praziqutnel (Chapter 175). Pp 1169 – 1171. In: Hunter's Tropical Medicine and Emerging Infectious Diseases. Tenth Edition, Ryan ET, Hill DR, Solomon T, Aronson NE, Endy TP **(eds). New York, Elsevier, Inc. 2020.**

14. Wang H, Fang ZZ, Zheng Y, Zhou K, Hu C, Krausz KW, Sun D, Idle JR, Gonzalez FJ. Metabolic profiling of praziquantel enantiomers. Biochem Pharmacol. 2014; 90(2):166-78. https://doi: 10.1016/j.bcp.2014.05.001.

15. Martinez MN, Mochel JP, Neuhoff S, Pade D. Comparison of canine and human physiological factors: understanding interspecies differences that impact drug pharmacokinetics. AAPS J. 2021; 23(3):59. https://doi: 10.1208/s12248-021-00590-0.

16. Hayeshi R, Masimirembwa C, Mukanganyama S, Ungell AL. The potential inhibitory effect of antiparasitic drugs and natural products on P-glycoprotein mediated efflux. Eur J Pharm Sci. 2006; 29(1):70-81. https://doi: 10.1016/j.ejps.2006.05.009.
